# Supplementary material for: Arthritogenic Alphavirus Vaccines: Serogrouping Versus Cross-Protection in Mouse Models
Source: Vaccines (Basel). 2020 May 5;8(2):209. doi: 10.3390/vaccines8020209 (PMC7349283; doi:10.3390/vaccines8020209)
Supplement: Supplementary file 1 [file vaccines-08-00209-s001.pdf]

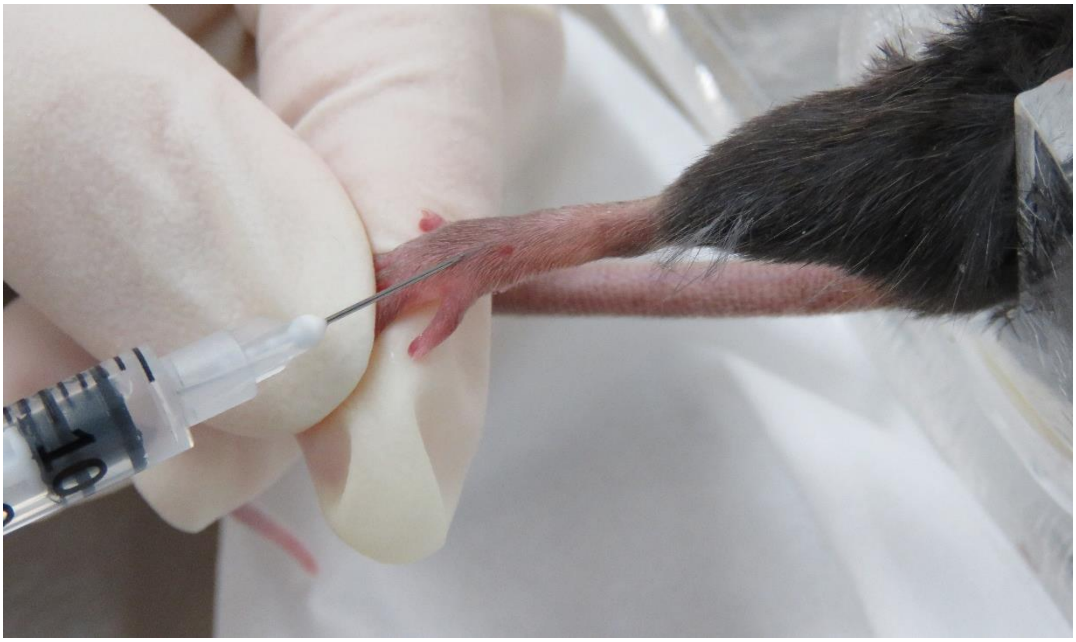

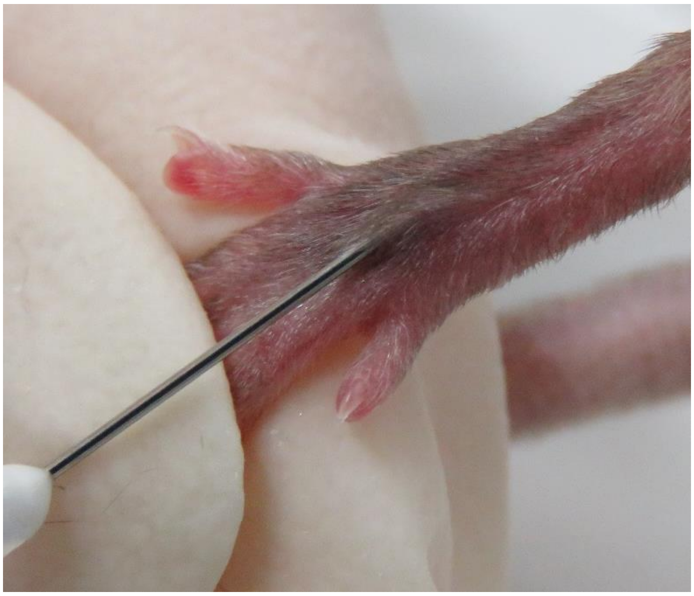

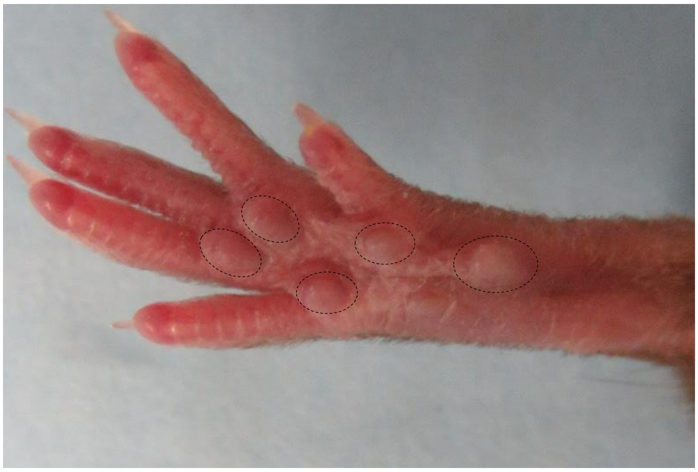

Supplementary Fig. 1. Injection of alphaviruses into hind feet. An insulin syringe is used to inject 40 µl of 10^4 $CCID_{50}$ of alphavirus in medium s.c. into the top/side of each hind foot. The mouse is held in a restrainer (cut from a 50 ml mounted syringe), with the foot pulled through a slit cut in the end of the restrainer.

The bottom of the foot (walking surface) is not injected for ethical reasons as this can cause discomfort and gait abnormalities. Footpads can be defined as the keratinized pads on which mice walk (walking pads) (bottom image, dashed ovals); no injection of these footpads is undertaken.

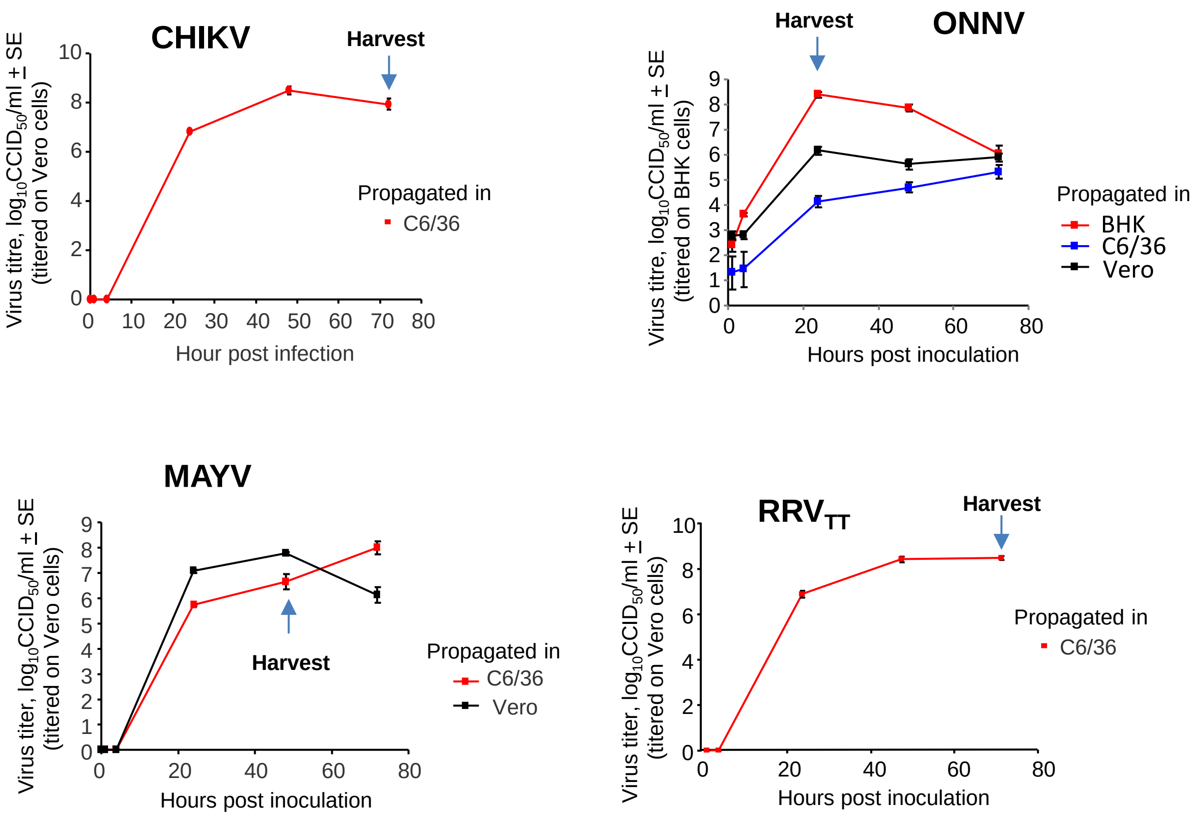

Supplementary Fig. 2. Growth of alphavirus stocks after infection at MOI≈0.01. Titres determined triplicate.

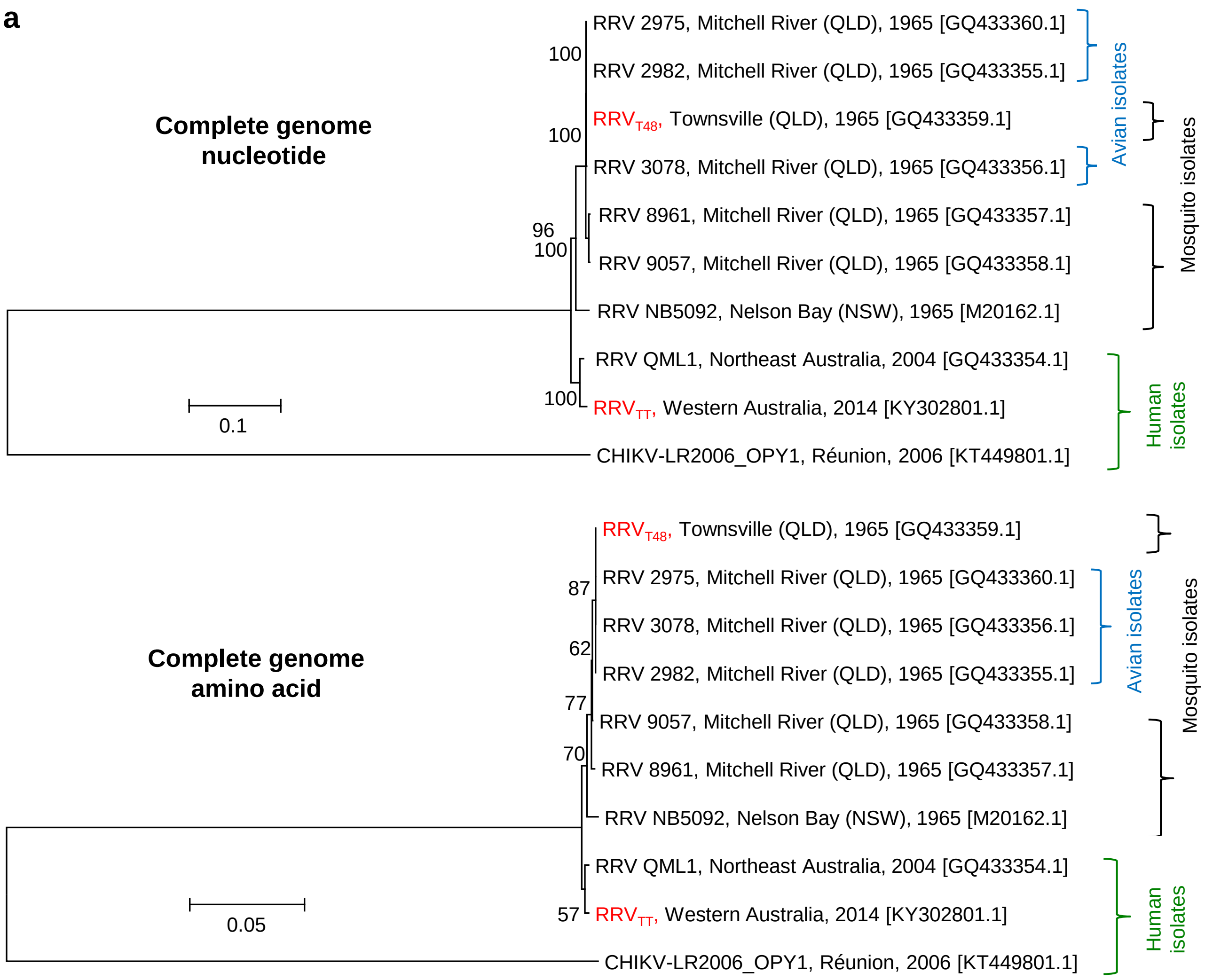

Supplementary Fig. 3. Comparisons between RRV_{TT} and RRV_{T48}. a. Phylogenetic trees of RRV sequences**.** The nucleotide sequence phylogenetic tree was constructed using maximum likelihood algorithms from complete open reading frame nucleotide sequences. Scale bar gives the substitutions per site and bootstrap values are given as a percentage of 1000 replicates. GenBank accession numbers are provided in square brackets. The amino acid phylogenetic tree was constructed in the same way and used the complete amino acid sequences for the indicated RRV isolates. For both phylogenetic trees, RRV_{TT} clusters with another human isolate, RRV QML1 (98% nucleotide identity), and is relatively distant from RRV_{T48} (96% nucleotide identity). RRV_{TT} was passaged once on C6/36 and once on Vero cells.

b

	Conservative differences			
Gene	RRV_{T48}	RRV_{TT}	Protein position	n
nsP1	M	L	239	3
	I	V	278	
	E	D	502	
nsP2	A	T	545	3
	R	K	565	
	E	D	650	
nsP3	V	I	1172	7
	S	T	1684	
	H	R	1699	
	R	H	1765	
	V	I	1840	
	I	V	1842	
	S	P	1850	
nsP4	V	I	2308	2
	V	I	2329	
C	T	A	35	1
E2	I	L	400	2
	R	K	585	
E1	V	I	928	4
	L	M	1129	
	S	G	1131	
	V	L	1142	

c

	Non-conservative differences			
Gene	RRV_{T48}	RRV_{TT}	Protein position	n
nsP1	V	A	186	3
	I	T	248	
	R	Q	522	
nsP2	M	T	674	3
	S	L	1114	
	Q	R	1153	
nsP3	T	M	1662	7
	T	I	1739	
	K	T	1754	
	T	K	1759	
	C	R	1788	
	K	E	1825	
	V	A	1826	
nsP4	V	A	1899	5
	L	Q	1910	
	Q	L	1918	
	T	I	1956	
	L	F	2093	
C	V	A	34	1
E3	R	G	329	1
6K	F	I	801	2
	A	V	813	
E1	S	L	935	2
	V	A	1120	

d

Specificity	RRV E2	RRV	Sindbis virus	Barmah Forest virus	Pan flavivirus
Mclab	G8	$B8_2A_2$	2F2	10E10-C2	4G2
RRV_{T48}	+	+	-	-	-
RRV_{TT}	+	+	-	-	-
BFV	-	-	-	+	-
SINV	-	-	+	-	-
KUNV	-	-	-	-	+

e

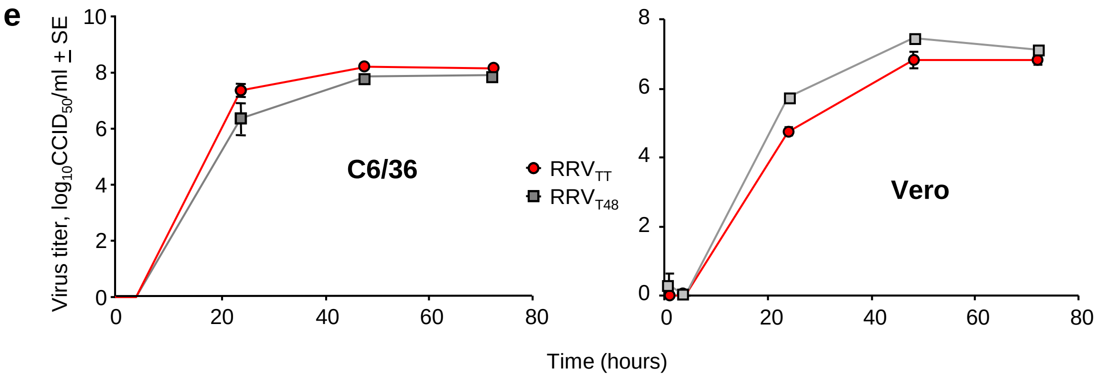

Supplementary Fig. 3. **b** Conservative amino acid differences (as defined by Miyata et al., 1979. J Mol Evol 12:219-36) between RRV_{TT} and RRV_{T48}. **c** Non-conservative amino acid changes between RRV_{TT} and RRV_{T48}. **d** Binding of monoclonal antibodies in fixed cell ELISAs to Sindbis virus (SINV), Barmah Forest virus (BFV) and WNV Kunjin strain (KUNV) using a panel of monoclonal antibodies; RRV-specific G8, RRV-specific B82A2, SINV-specific 2F2 (kindly provided by Dr G Burgess, James Cook University), BFV-specific 10E10-C2 (kindly provided by Dr Burgess) and the pan-flavivirus 4G2 (ATCC# HB-112). **e** Replication of RRV_{TT} and RRV_{T48} after infection of C6/36 or Vero cells at MOI=0.01 for 1 hour followed by washing. Each data point is the mean of 3 wells (tested using 10 replicates) from 2 independent experiments (n=6).

CHIKV infections

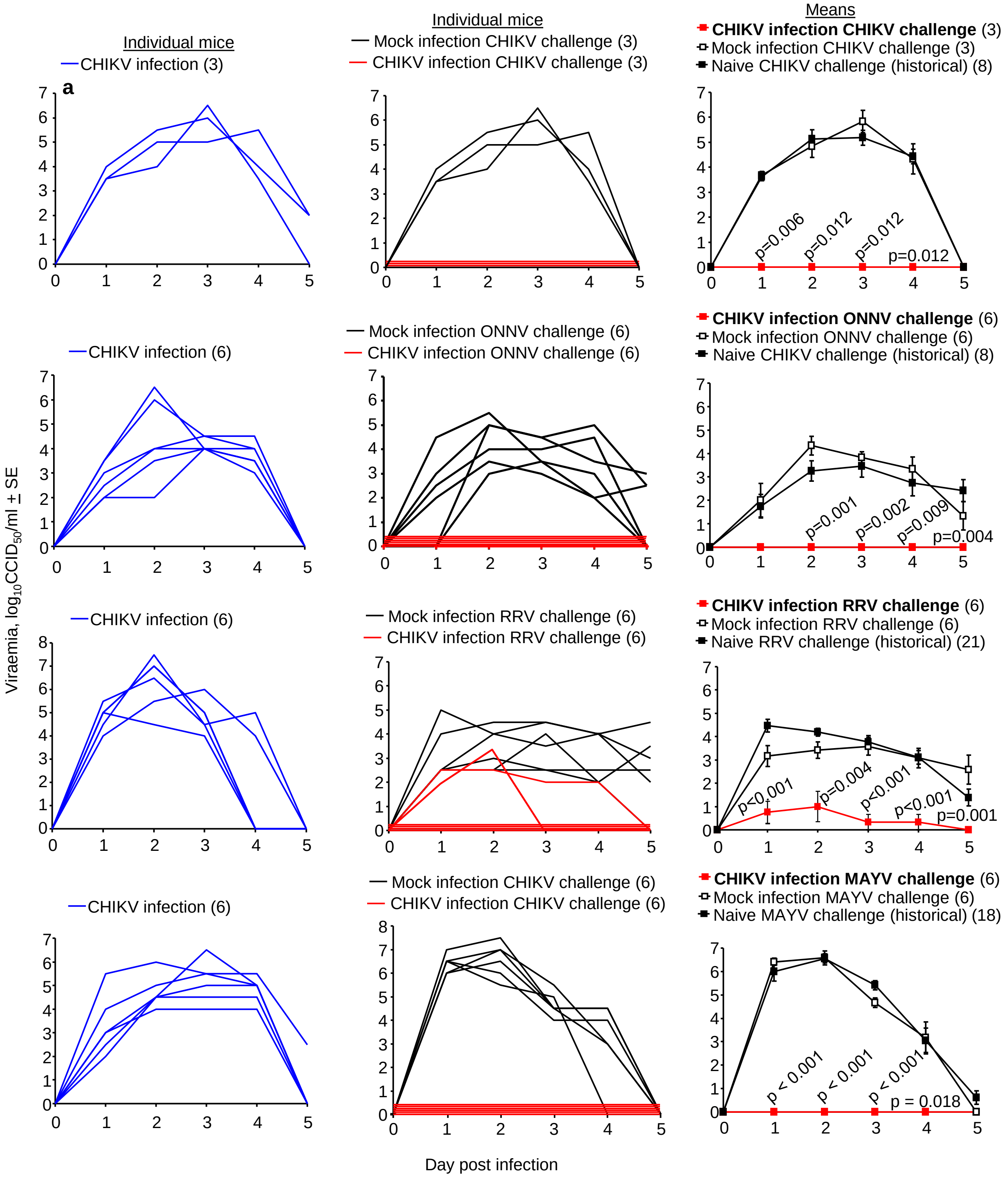

Supplementary Fig. 4a. **Individual mouse data for Fig. 2**. CHIKV infections and CHIKV, ONNV, RRV and MAYV challenge. Number in brackets represent the number of mice per group. Statistics by Kolmogorov-Smirnov tests taking mock and naive together as 1 group.

b

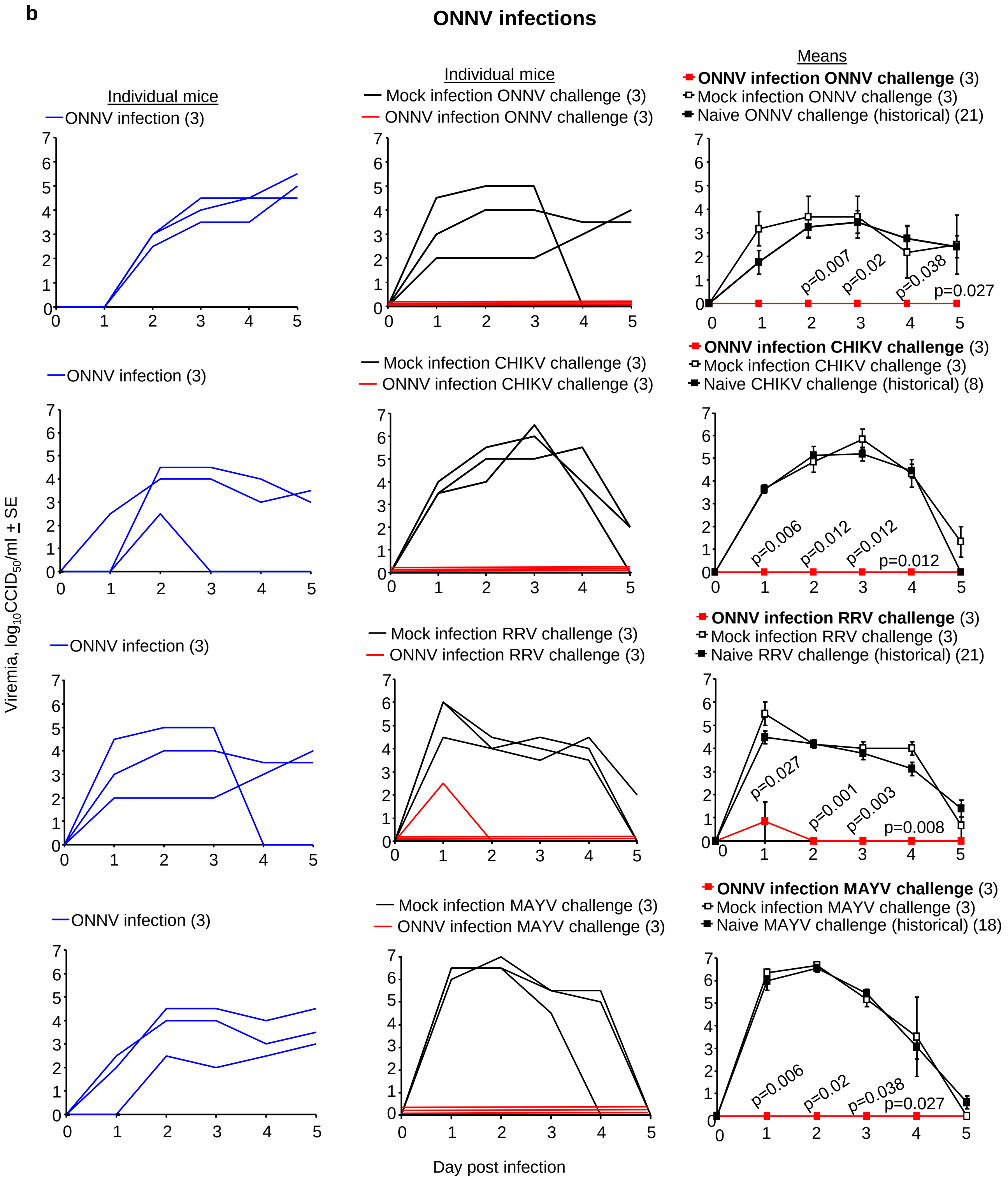

Supplementary Fig. 4b. Individual mouse data for Fig. 2. ONNV infections and CHIKV, ONNV, RRV and MAYV challenge. Number in brackets represent the number of mice per group. Statistics by Kolmogorov-Smirnov tests taking mock and naive together as 1 group.

c

RRV infections

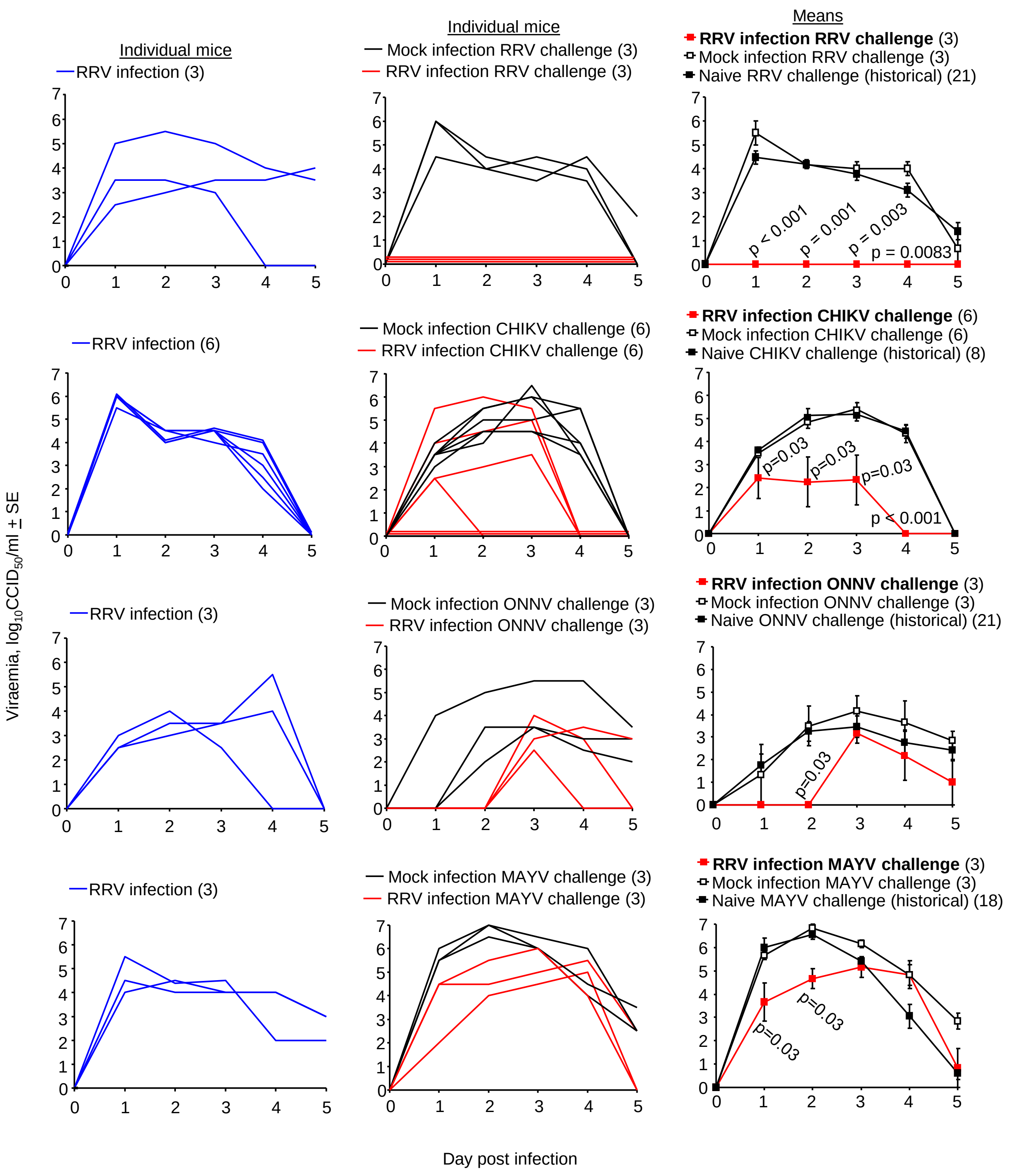

Supplementary Fig. 4c. Individual mouse data for Fig. 2. RRV infections and CHIKV, ONNV, RRV and MAYV challenge. Number in brackets represent the number of mice per group. Statistics by Kolmogorov-Smirnov tests taking mock and naive together as 1 group.

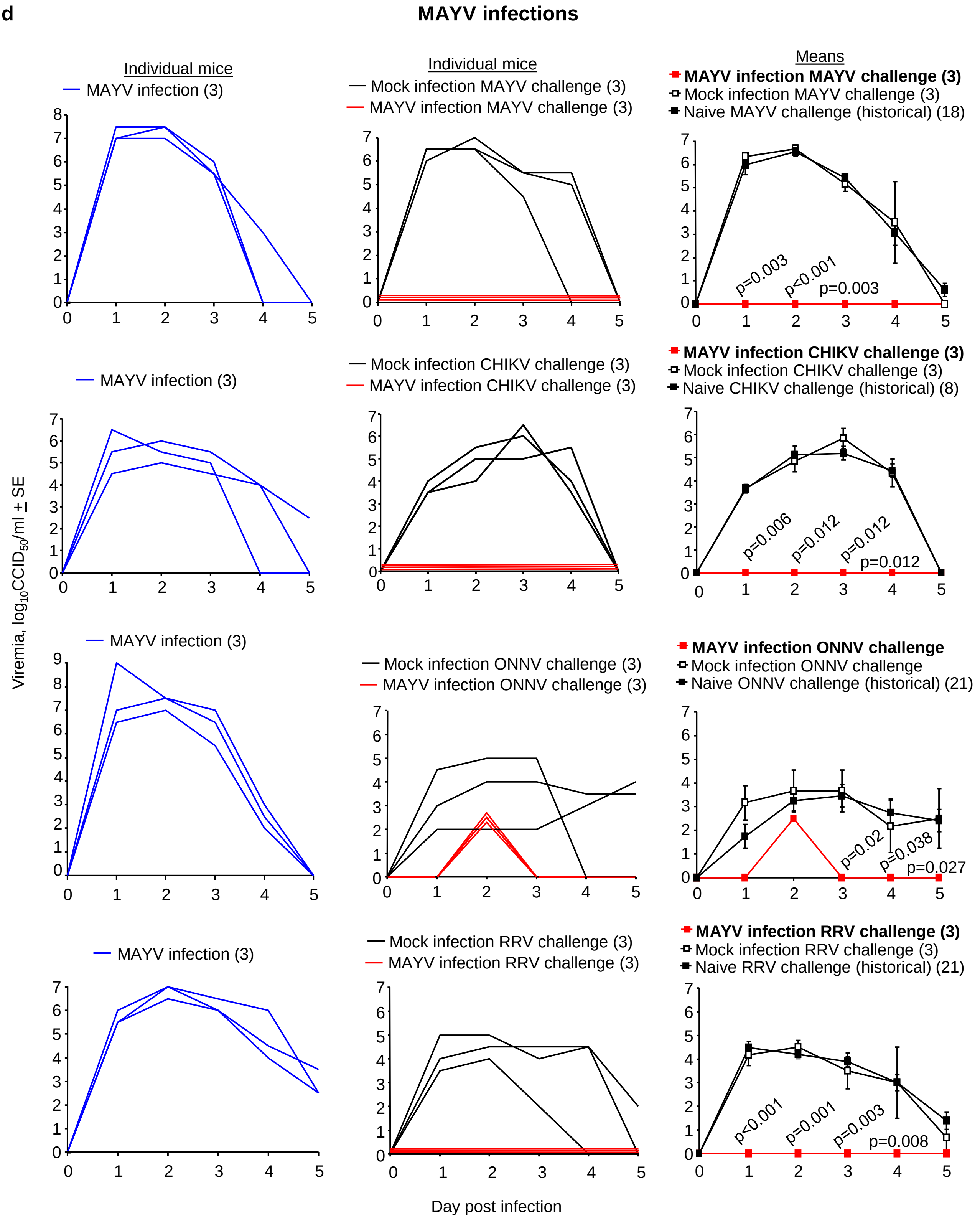

Supplementary Fig. 4d. Individual mouse data for Fig. 2. MAYV infections and CHIKV, ONNV, RRV and MAYV challenge. Number in brackets represent the number of mice per group. Statistics by Kolmogorov-Smirnov tests taking mock and naive together as 1 group.

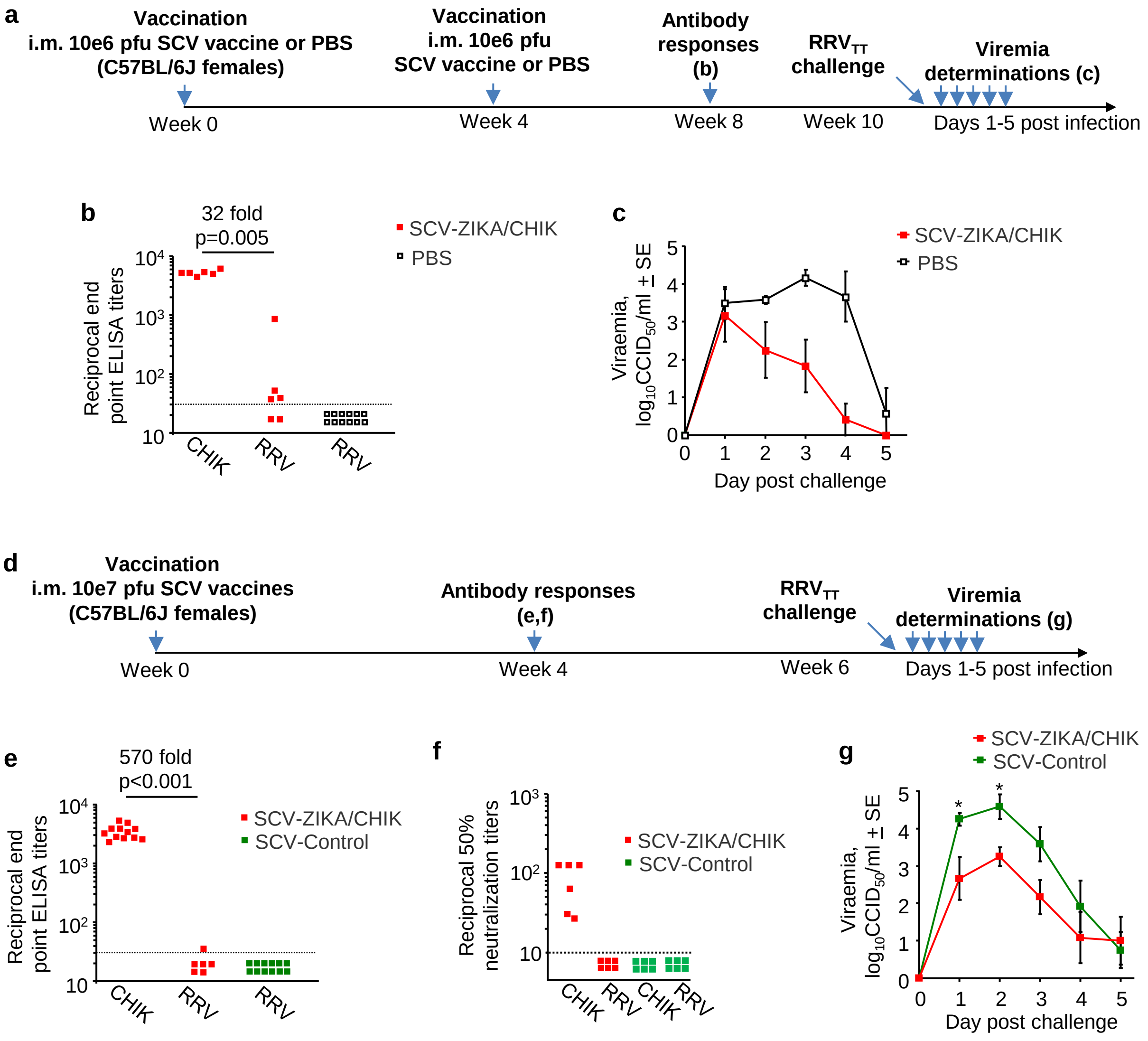

Supplementary Fig. 5. Protection against RRV_{TT} with difference doses/schedules of SCV-ZIKA/CHIK vaccination. Cross-reactive antibody responses and protection against RRV_{TT} afforded by vaccination with two shots of 10e6 pfu or 1 shot of 10e7 pfu of SCV-ZIKA/CHIK. **a** Time line of vaccinations with 10e6 pfu of SCV-ZIKA/CHIK, antibody response determinations and challenge. **b** End point ELISA titers after two vaccinations with 10e6 pfu of SCV-ZIKA/CHIK. Statistics by Kolmogorov-Smirnov test. **c** RRV_{TT} viremia post challenge with RRV_{TT} (n=6 mice per group). No significant difference in viraemia on any given day; Kolmogorov-Smirnov tests. **d** Time line of vaccination with 10e7 pfu of SCV-ZIKA/CHIK, antibody response determinations and challenge. **e** End point ELISA titers after one vaccination with 10e7 pfu of SCV-ZIKA/CHIK. **f** Neutralizing antibody titers after one vaccination with 10e7 pfu of SCV-ZIKA/CHIK. **g** RRV_{TT} viremia post challenge with RRV_{TT} (n=6 mice per group). *Significant differences in viremia on days 1 and 2, p=0.015, Kolmogorov-Smirnov tests.

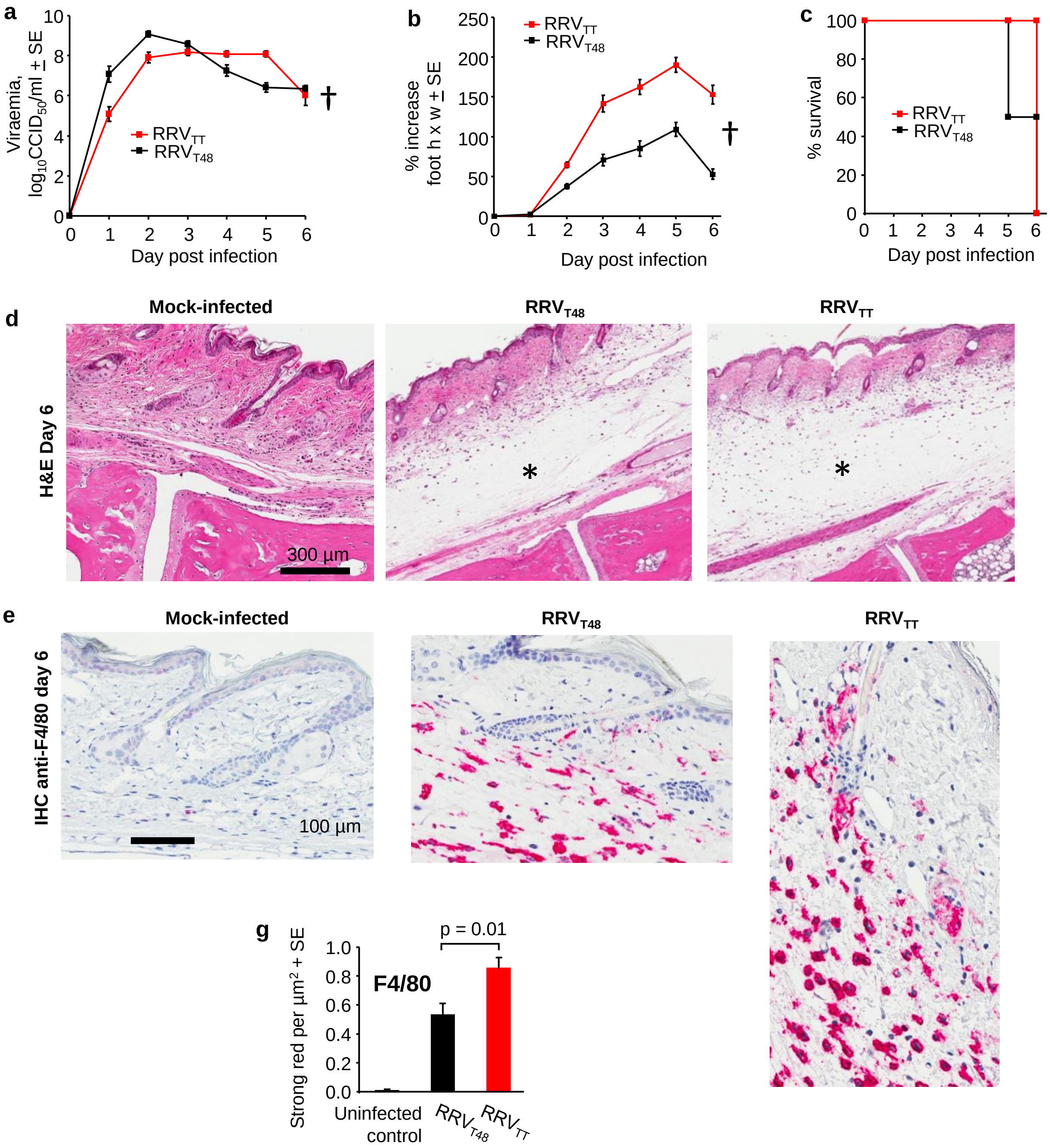

Supplementary Fig. 6. **RRV infection of IRF3/7$^{-/-}$ mice**. **a** Viremia post infection with RRV_{TT} and RRV_{T48} (n=6 per group) (cross – euthanasia). **b** Foot swelling (n=12 feet from 6 mice). **c** Survival (n=6 per group). **d** H&E staining of mock-infected and RRV infected mice showing subcutaneous edema (*). **e** IHC for anti-F4/80 staining showing subcutaneous monocyte/macrophage infiltration (red staining). **g** Aperio Positive Pixel Count determination of anti-F4/80 staining (7-9 whole foot sections from 3 feet from 3 mice per group; statistics by t test).

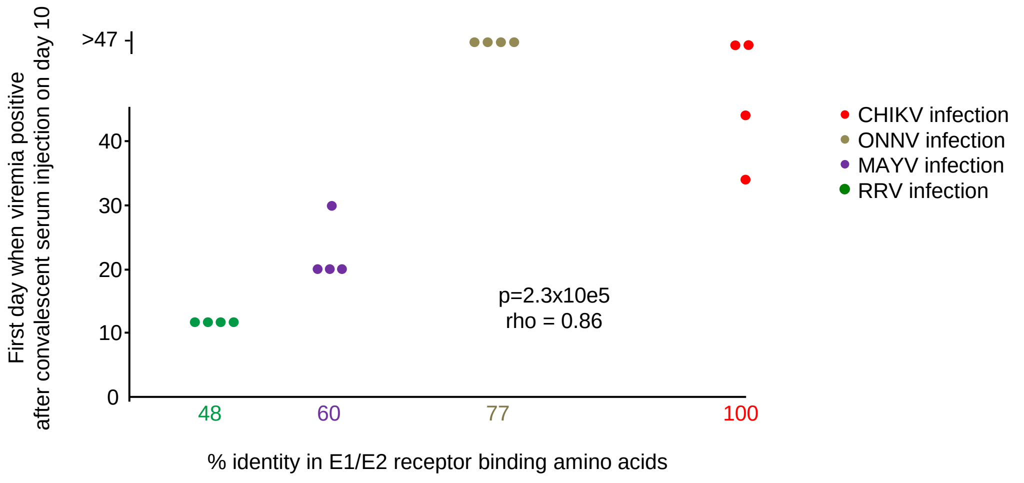

Supplementary Fig. 7. Virus reappearance in Rag1$^{-/-}$ mice correlates with E1/E2 identity. Using the data provided in Fig. 7b, the first day when viremia was detectable (after convalescent CHIKV serum was injected on day 10 into Rag1$^{-/-}$ mice persistently infected with the indicated virus) was plotted against the level of receptor contact residue identity relative to CHIKV shown in Fig. 3b. Thus the four Rag1$^{-/-}$ mice persistently infected with RRV$_{TT}$ were viremia positive on day 11 (one day after serum injection), and RRV$_{TT}$ showed 48% amino acid identity with CHIKV. All the Rag1$^{-/-}$ mice persistently infected with ONNV were viremia negative on day 47 (thus deemed positive on a day >47) and showed 77% amino acid identity with CHIKV. Statistics by Spearman rank correlation, p and rho indicated.

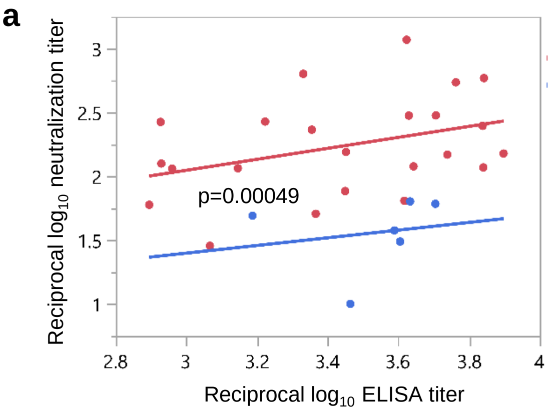

Supplementary Fig. 8. Statistical comparisons of neutralizing and ELISA antibodies.

a After vaccination with 10e6 pfu of SCV-ZIKA/CHIK, the ELISA and neutralizing antibody titers against CHIKV and ONNV were determined. After SCV-ZIKA/CHIK vaccination a significantly lower proportion of ELISA-detectable cross-reactive anti-ONNV antibodies were able to mediate ONNV neutralization (blue dots), when compared with ELISA-detectable anti-CHIKV antibodies able to mediate CHIKV neutralization (red dots). Thus for a given ELISA titer, neutralization titers were on average 0.5–1 logs higher than cross-neutralization titers. Each dot represents data from one mouse.

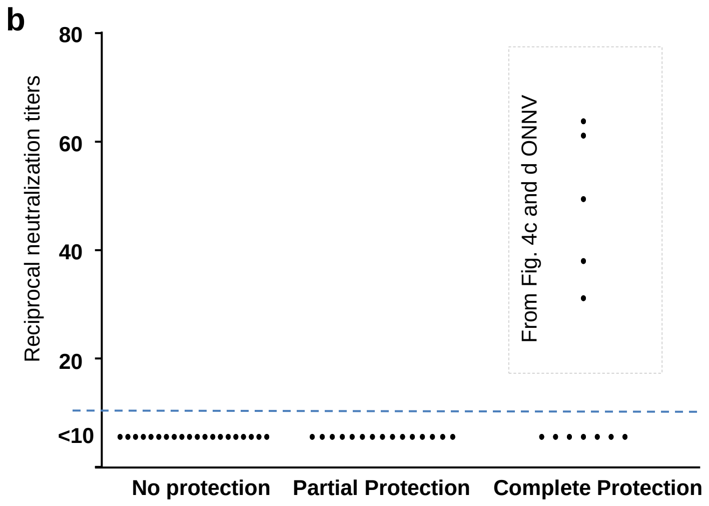

b Taking all SCV-ZIKA/CHIK and JEV/GETV vaccine data together, with each dot representing 1 mouse. All reciprocal neutralization titers >10 provided complete protection against viremia. All mice showing No protection or Partial protection had reciprocal neutralization titers <10.

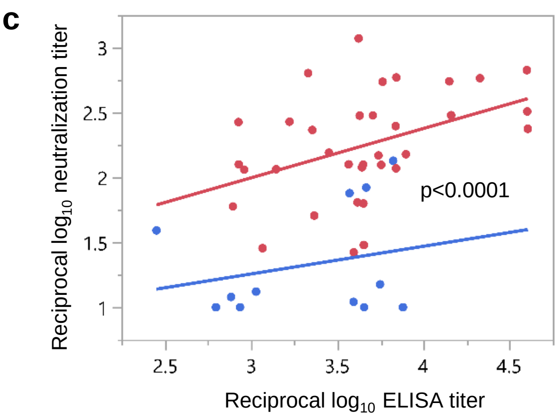

c. A significantly higher proportion of ELISA-detectable anti-CHIKV antibodies were able to mediate CHIKV neutralization after SCV-ZIKA/CHIK vaccination (all doses) (red dots), when compared with ELISA-detectable anti-GETV antibodies able to mediate GETV neutralization after JEV/GETV vaccination (blue dots).

Statistics for a and c by Parallelism of regression lines test using JMP Pro version 15.0.0.

Use of Rag$^{-/-}$ mice to evaluate antiviral biologics and drugs.

CHIKV-specific IgM has been shown to have neutralizing activity [1,2]. Here we illustrate the use the Rag$^{-/-}$ model to test the activity of an E1/E2 specific, RRV-specific IgM monoclonal antibody, 37B2, which has established neutralizing activity *in vitro* [3]. Rag2$^{-/-}$ mice, with a persistent RRV_{TT} viremia, injected with 37B2 (but not a control monoclonal IgM 3G1.1) showed a significant transient ≈4 log drop in viremia (a). This result supports the view that alphavirus-specific IgM antibodies can mediate some anti-viral activity *in vivo*, and also illustrates the utility of this model for testing anti-alphaviral biologics.

Ribavirin, a guanosine analogue used in the treatment of hepatitis C and viral hemorrhagic fevers, has been reported to have *in vitro* activity against alphaviruses [4-6]. Persistently infected Rag2$^{-/-}$ mice were used to test the activity of ribavirin against RRV_{TT} infections. A dose of 100 mg/kg of ribavirin for five consecutive days had no significant effect on the viremia (b), suggesting ribavirin has limited utility as an anti-viral agent for RRV.

1. Chua, C.L.; Sam, I.C.; Chiam, C.W.; Chan, Y.F. The neutralizing role of IgM during early Chikungunya virus infection. *PLoS One* **2017**, *12*, e0171989,
2. Lam, S.; Nyo, M.; Phuektes, P.; Yew, C.W.; Tan, Y.J.; Chu, J.J. A potent neutralizing IgM mAb targeting the N218 epitope on E2 protein protects against Chikungunya virus pathogenesis. *MAbs* **2015**, *7*, 1178-1194.
3. Oliveira, N.M. Analysis of Ross River virus using monoclonal and polyclonal antibodies with applications in diagnostic and surveillance techniques. *PhD Thesis. University of Western Australia.* http://bit.ly/2kKLq41 **1998**.
4. Ravichandran, R.; Manian, M. Ribavirin therapy for chikungunya arthritis. *J Infect Dev Ctries* **2008**, *2*, 140-142.
5. Rothan, H.A.; Bahrani, H.; Mohamed, Z.; Teoh, T.C.; Shankar, E.M.; Rahman, N.A.; Yusof, R. A combination of doxycycline and ribavirin alleviated chikungunya infection. *PLoS One* **2015**, *10*, e0126360.
6. Dash, P.K.; Agarwal, A.; Sharma, S.; Saha, A.; Joshi, G.; Gopalan, N.; Sukumaran, D.; Parida, M.M. Development of a SYBR green I-based quantitative RT-PCR for Ross River virus: Application in vector competence studies and antiviral drug evaluation. *J Virol Methods* **2016**, *234*, 107-114

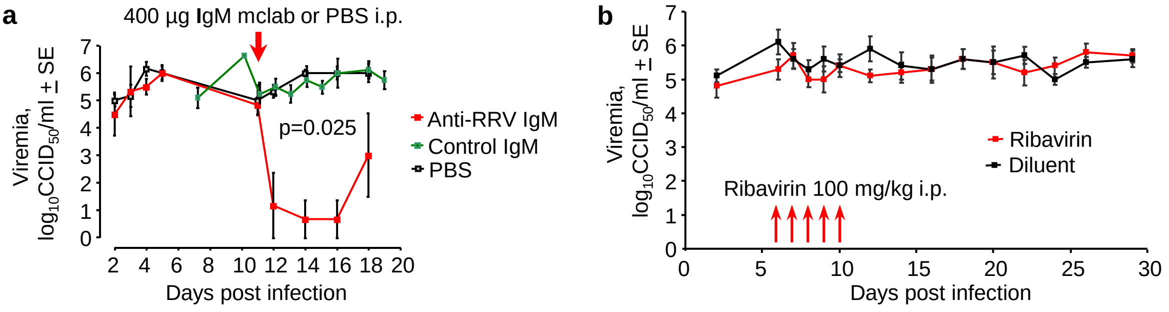

Supplementary Fig. 9. Anti-RRV_{TT} activity of IgM and ribavirin. **a** Purified anti-RRV IgM monoclonal antibody (37B2) or a control IgM monoclonal (3G1.1) or PBS were injected i.p. into Rag2-/- mice (red arrow) with persistent RRV_{TT} viremias (n=3, 4 and 4 mice, respectively). Treatment with 37B2 resulted in a significant drop in viremia on days 12, 14, 16 and 18 (p=0.025 for each day, Kolmogorov-Smirnov tests, comparing 37B2, n=3, with 3G1.1 and PBS taken together, n=8). **b** Ribavirin was used to treat Rag2-/- mice (100 mg/kg, red arrows) with persistent RRV_{TT} viremias.
